# Correlation of Baba’s diabetic neuropathy classification with various diabetes-related complications

**DOI:** 10.3389/fendo.2022.1054934

**Published:** 2022-10-27

**Authors:** Yuichiro Iwamoto, Shuhei Nakanishi, Takashi Itoh, Erina Nakao, Toshitomo Sugisaki, Takashi Kusano, Mana Ohnishi, Haruka Takenouchi, Hideyuki Iwamoto, Junpei Sanada, Yoshiro Fushimi, Yukino Katakura, Shoji Hemmi, Tomohiko Kimura, Fuminori Tatsumi, Masashi Shimoda, Tomoatsu Mune, Kohei Kaku, Hideaki Kaneto

**Affiliations:** ^1^ Division of Diabetes, Metabolism and Endocrinology, Kawasaki Medical School, Kurashiki, Japan; ^2^ Division of Neurology, Kawasaki Medical School, Kurashiki, Japan

**Keywords:** diabetes mellitus, diabetic polyneuropathy (DPN), complication, nerve conduction test, intima and media thickness

## Abstract

It is known that Baba’s diabetic neuropathy classification (BDC) is useful in quantitative evaluation of Diabetic polyneuropathy (DPN). In this study, we aimed to investigate the possible association between BDC and various diabetic microvascular and macrovascular complications in patients whose neuropathy was evaluated with BDC. As the results, BDC was significantly correlated with the severity of diabetic retinopathy and nephropathy. BDC was also significantly correlated with history of myocardial infarction or cerebral infarction, carotid IMT, and ABI. These data suggest that BDC may be useful in predicting the presence of various diabetic microvascular and macrovascular complications. The data also support the idea that we should perform further investigation of other diabetes-related complications in patients with severe DPN.

## Introduction

Diabetic polyneuropathy (DPN), a microvascular complication of diabetes, usually occurs in a relatively early stage of diabetes compared to other microvascular complications (1). DPN is characterized by polyneuropathy and autonomic neuropathy, with negative (e.g., hypoesthesia) and/or positive (e.g., pain) symptoms that often reduce patients’ quality of life (2) (3). The diagnosis is often delayed and clinically problematic in cases with only negative symptoms or mild impairment compared to those with positive symptoms. The golden standard in the diagnosis of diabetic polyneuropathy is nerve conduction study (NCS) (4). Baba’s diabetic neuropathy classification (BDC) has been proposed to evaluate nerve conduction velocity in subjects with DPN and is useful for quantitative evaluation of neuropathy (5). Indeed, it was reported that cardiovascular prognosis was significantly worsen in patients with moderate to severe DPN assessed with this classification (6).

We use this method when diabetic patients are admitted to our hospital to accurately assess neuropathy, and although it has been suggested that DPN is associated with pathological progression of atherosclerotic lesions, there are few reports on the association between BDC and microvascular and macrovascular complications of diabetes mellitus. In this study, we aimed to investigate the possible association between BDC and various diabetic microvascular and macrovascular complications in patients whose neuropathy was evaluated with BDC.

## Materials and methods

### Study population and patient preparation

**Figure 1:**
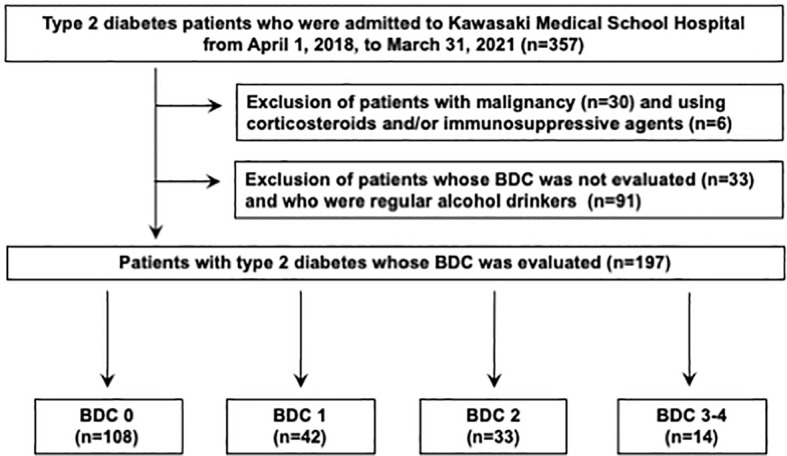
The flowchart of the participants and exclusions in this study.

**Figure 2:**
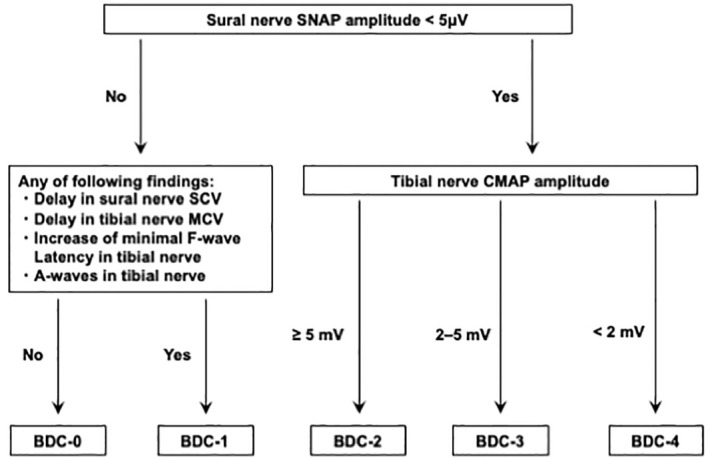
How to evaluate Baba’s diabetic classification by nerve conduction studies.

This was a single-center, retrospective, cross-sectional study of patients with type 2 diabetes. A total of 357 patients eligible for the present study who were admitted to our hospital for treatment of type 2 diabetes at Kawasaki Medical School Hospital from April 1st, 2018, to March 31st, 2021. The Institutional Review Board of Kawasaki Medical School (No. 5594-00) approved the study protocol, including opt-out informed consent. This study was conducted by the principles of the Declaration of Helsinki. The flow of participants in this study is shown in **Figure 1**. Among the 357 eligible adults with type 2 diabetes mellitus, 30 participants with malignancy and 6 participants who were taking corticosteroids and/or immunosuppressive drugs were excluded from the study. Subsequently, 33 participants whose nerve conduction velocity was not assessed and 91 participants who were regular alcohol drinkers were excluded. Finally, 197 type 2 diabetics whose nerve conduction velocity was assessed using BDC were included. The definition of BDC used in this study is shown in **Figure 2**: sensory nerve conduction velocity (SCV) and sensory nerve action potential (SNAP) in the sural nerve and motor nerve conduction velocity (MCV) and compound muscle action potential (CMAP) in the tibial nerve were evaluated (5, 6). Based on the results, the patients were grouped into BDC 0 (normal), BDC 1 (mild), BDC 2 (moderate), BDC 3 (moderate to severe) and BDC 4 (severe). BDC3 and BDC4 are both clinically severely neurologically dysfunctional conditions and were analyzed as the same group in this study. Consequently, 108 patients were grouped in BDC 0, 42 in BDC 1, 33 in BDC 2 and 14 in BDC 3-4.

### Method of examination

Study participants’ age, duration of diabetes, history of smoking, history of alcohol consumption, and current medications were obtained at the time of admission. Cases who consumed more than 20 g/day of ethanol three or more times per week were excluded from the study. Smokers were divided into current smoker, past smoker, and never smoker. All patients were asked on admission by their physician whether they had an old cerebral infarction and an old myocardial infarction. Body weight, body mass index (BMI), abdominal circumference, grip strength, blood pressure, and pulse rate were measured upon admission. Blood glucose, HbA1c, lipids, and liver and kidney function data were assessed by fasting blood tests on the day following admission.

The following is a brief description of the complications of diabetes mellitus and how to test them. NCS was performed by skilled technicians using MEB-2306 or MEB-2312 (NIHON KOHDEN Corp., Tokyo). NCS was performed in the upper and lower extremities on one side and, in the presence of DPN symptoms, on the more symptomatic side. The data obtained were analyzed by a neurologist. Modified Davis staging was used to evaluate diabetic retinopathy (7), and ophthalmoscopy was used to classify into non-diabetic retinopathy (NDR), simple diabetic retinopathy (SDR), pre-proliferative retinopathy (PPDR), and proliferative diabetic retinopathy (PDR). SDR was diagnosed when retinal hemorrhage, retinal capillary varicoceles, and hard leukocoria were observed; PPDR was diagnosed when the above findings plus soft leukocoria were observed; PDR was diagnosed when neovascularization, vitreous hemorrhage, and tractional retinal detachment were observed in addition to the above findings. Diabetic nephropathy was evaluated by dividing the patients into 5 stages based on eGFR and albuminuria according to the disease stage in the Japan Diabetes Society as follows: Stage 1 was defined as eGFR >30 mL/min/1.73 m^2^ and urinary albumin <30 mg/gCr; Stage 2 was defined as eGFR >30 mL/min/1.73 m^2^ and urinary albumin; Stage 3: eGFR ≥ 30 mL/min/1.73 m^2^ and urinary albumin ≥ 300 mg/gCr, and Stage 4: eGFR < 30 mL/min/1.73 m^2^. Referring to the Current Chronic Kidney Disease (CKD) Nomenclature Used by KDIGO, participants were categorized by eGFR as follows: G1 - >90mL/min/1.73m^2^; G2 - 60-89mL/min/1.73m^2^; G3a - 45-59 mL/min/1.73 m^2^; G3b - 30-44 mL/min/1.73 m^2^; G4 - 15-29 mL/min/1.73 m^2^. Participants were also categorized by creatinine-converted albuminuria as follows: A1 - <30 mg/gCr; A2 - 30-300 mg/gCr; A3 - >300 mg/gCr. Participants in this study did not include dialysis patients. CVR-R was evaluated using a twelve-lead electrocardiogram measuring device. CVR-R at rest was calculated by measuring the interval between the apexes of the R wave in 100 consecutive heartbeats in the resting supine position. CVR-R at deep breath was calculated from the interval variability of the R wave of 100 consecutive heartbeats during deep breathing. Three patients with atrial fibrillation were excluded when assessing CVR-R because it is impossible to assess CV-RR in these patients. For vibration sensation, a C128 tuning fork was applied to the endocondyle of the tibia, the vibration sensing time was measured on both sides, and the average value was calculated. Carotid IMT was assessed by an ultrasound technician with ultrasonographic equipment, Aplio series (CANON MEDICAL SYSTEMS, Tochigi, Japan). The carotid IMT was evaluated in the common carotid artery wall 10 mm centrally from the carotid sinus. Two IMT points were measured within a 10 mm area, and the mean value was defined as mean IMT. The maximum diameter in the same measurement range was defined as max IMT. Ankle brachial index (ABI) was calculated by measuring blood pressure at the bilateral upper arm and ankle in the supine position and by the ratio of the blood pressure at the higher upper arm to the blood pressure at the respective ankles on the right and left sides. In this study, the lower values of the left and right ABIs were included in the analysis.

### Statistical analysis

The primary endpoint was the correlation between BDC and atherosclerotic lesions, and secondary endpoints were diabetes-related parameters on admission and factors that could correlate with BDC. Analysis between BDC groups was performed by chi-square test and ANOVA. ANOVA adjusted for age, gender, disease duration, smoking history, HbA1c, BMI, hypercholesterolemia medication, and hypertension medication was used to evaluate mean IMT, max IMT, and ABI among BDC groups. *Post hoc* testing was performed by the Tukey method. Mean IMT, max IMT, and ABI were evaluated by natural logarithm. JMP (16.0.1) was used for analysis and EXCEL for Mac (16.58) for table creation.

## Results

### Clinical characteristics of study participants

The clinical characteristics of the patients in this study are summarized in **Table 1**. The age of all participants was 60.6 ± 15.1 years (mean ± standard deviation), HbA1c was 10.3 ± 2.3%, and duration of diabetes was 12.2 ± 11.7 years. BDC and vibration sensation, CVR-R at rest, and CVR-R at deep breathing were all significantly correlated (p<0.005, p<0.05, and p<0.05, respectively). Smoking history, BMI, blood pressure, and HbA1c were not significantly different in each group. Prevalence of previous myocardial infarction increased with increasing BDC grade (p<0.05). Compared to BDC-0, BDC-3/4 were older, had a longer history of diabetes, and were more likely to take insulin injections, alpha-glucosidase inhibitors, and medications for hypercholesterolemia and hypertension.

**Table 1:** Comparison of various parameters among patients grouped with Baba’s diabetic neuropathy classification (BDC).

Parameter	BDC-0 (n = 108)	BDC-1 (n = 42)	BDC-2 (n = 33)	BDC-3/4 (n = 14)
Male/female	51/57	27/15	18/15	9/5
Age (years) *	57.9 ± 16.0	63.6 ± 13.2	62.9 ± 13.1	67.5 ± 12.6
Duration of diabetes (years) *	9.9 ± 9.6	11.5 ± 11.1	14.5 ± 9.2	26.6 ± 20.1
Body weight (kg)	70.3 ± 17.5	70.5 ± 16.9	76.5 ± 21.1	67.8 ± 16.4
BMI (kg/m^2^)	27.2 ± 5.3	25.7 ± 4.7	29.3 ± 7.3	25.5 ± 4.5
Abdominal circumference (cm)	92.4 ± 13.2	91.4 ± 12.8	102.3 ± 19.3	92.9 ± 12.1
Grip strength (kg) *	26.9 ± 10.6	28.4 ± 11.2	25.5 ± 9.5	21.0 ± 8.4
Smoking history (current/past/never, %) *	42.9/28.6/28.5	24.2/12.1/63.7	34.1/19.5/46.4	26.2/11.2/62.6
History of old myocardial infarction (%) *	5.6	11.9	21.2	28.6
History of old cerebral infarction (%) *	3.8	0	0	28.6
Systolic blood pressure (mmHg)	127.8 ± 22.9	128.8 ± 20.3	127.1 ± 17.4	127.1 ± 16.6
Diastolic blood pressure (mmHg)	79.6 ± 12.9	77.3 ± 14.6	74.5 ± 12.0	74.8 ± 11.8
Pulse rate (beats/min)	85.8 ± 14.3	81.6 ± 13.3	78.3 ± 16.2	85.2 ± 13.9
Vibration sensation (sec) *	12.6 ± 0.8	11.5 ± 1.2	10.3 ± 1.2	9.3 ± 1.4
CVR-R at rest (%) *	3.13 ± 0.34	2.61 ± 0.49	3.05 ± 0.49	1.69 ± 0.57
CVR-R at deep breathing (%) *	5.04 ± 0.51	3.96 ± 0.74	4.70 ± 0.74	2.47 ± 0.87
Blood glucose (mg/dL)	164.2 ± 49.2	160.8 ± 50.8	159.1 ± 58.2	183.0 ± 32.6
HbA1c (%, NGSP)	10.1 ± 2.3	10.9 ± 2.7	10.0 ± 2.0	11.0 ± 1.8
Triglyceride (mg/dL) *	222.5 ± 287.1	160.2 ± 83.9	218.8 ± 212.1	133.89 ± 49.3
LDL-cholesterol (mg/dL) *	108.8 ± 33.3	117.1 ± 45.6	95.9 ± 28.3	86.8 ± 24.7
HDL-cholesterol (mg/dL)	42.2 ± 10.0	43.5 ± 11.2	42.1 ± 9.7	42.8 ± 5.4
AST (U/L)	34.6 ± 29.1	27.4 ± 17.2	31.9 ± 19.1	22.0 ± 4.1
ALT (U/L)	40.5 ± 32.8	29.8 ± 16.2	30.7 ± 19.8	22.8 ± 5.9
Urea nitrogen (mg/dL) *	14.8 ± 4.1	17.2 ± 4.8	16.1 ± 6.2	28.5 ± 12.2
Creatinine (mg/dL) *	0.73 ± 0.23	0.82 ± 0.35	0.84 ± 0.20	1.15 ± 0.43
Percentage of drug at time of admission				
Insulin injections *	29.6	26.2	36.4	71.4
GLP-1 receptor agonist	15.7	21.4	18.2	35.7
Sulfonylurea	5.6	19.1	12.1	14.3
Glinide	7.3	2.4	9.1	7.1
Dipeptidyl peptidase-4 inhibitor	47.2	38.1	36.4	28.6
Biguanide	50.9	50.0	39.4	42.9
Thiazolidine	12.0	9.5	18.2	0
Alpha glucosidase inhibitor *	2.8	11.9	12.1	21.4
Sodium glucose co-transporter 2 inhibitor	33.3	31.0	36.4	28.6
Hypercholesterolemia medications *	43.5	38.1	54.5	64.3
Hypertension medications *	38.9	50.0	57.6	71.4

Data are presented as mean ± standard deviation. BDC, Baba’s diabetic neuropathy classification; BMI, body mass index.

*: P < 0.05 with chi-square test and ANOVA.

### Correlation between BDC and diabetes microvascular complications

**Figure 3:**
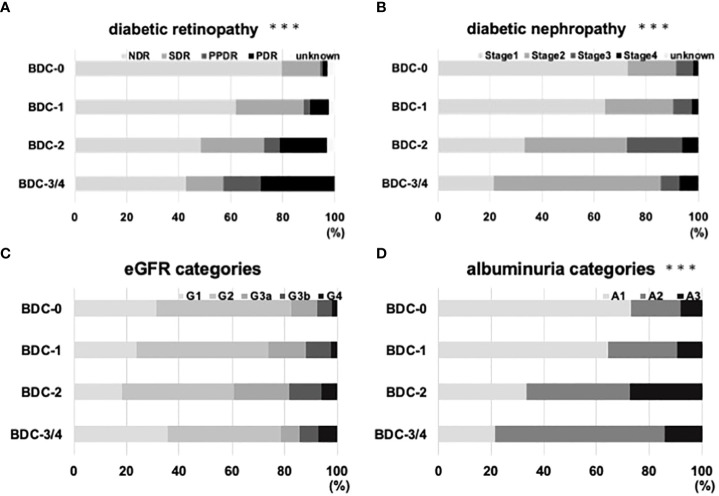
Relationship of Baba’s diabetic neuropathy classification (BDC) with diabetic microvascular complications. **(A)** Correlation between BDC and severity of diabetic retinopathy. **(B)** Correlation between BDC and severity of diabetic nephropathy. **(C)** Correlation between BDC and eGFR categories. **(D)** Correlation of albuminuria categories. ***P<0.0005 with chi-square test.

The correlation between BDC and diabetic retinopathy is shown in **Figure 3A**. the percentage of patients with retinopathy with SDR or higher in the modified Davis staging was 20.4% in BDC-0, 38.1% in BDC-1, 51.5% in BDC-2 and 57.1% in BDC-3/4. The correlation between BDC and diabetic nephropathy is shown in **Figure 3B**. 26.9% of BDC-0, 35.7% of BDC-1, 66.7% of BDC-2, and 78.6% of BDC-3/4 had Stage 2 or higher (p<0.0005, respectively). In **Figure 3C**, the correlation between eGFR categories and BDC was evaluated and there were no significant differences among the groups. On the other hand, BDC and severity of albuminuria categories were significantly correlated (**Figure 3D**).

### Correlation of BDC with atherosclerosis

**Figure 4:**
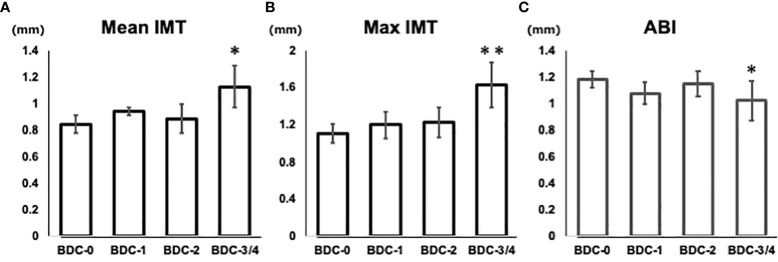
Relationship of Baba’s diabetic neuropathy classification (BDC) with arteriosclerosis. **(A)** Correlation between BDC and mean IMT. **(B)** Correlation between BDC and max IMT. **(C)** Correlation between BDC and ABI. Data are presented as mean and standard deviation (SD). *P<0.05, **P<0.005 with ANOVA adjusted for age, gender, duration of disease, smoking history, HbA1c, BMI, hyperlipidemia medications, and hypertension medications.

The correlation between BDC and carotid intima-media thickness (IMT) and ankle-brachial index (ABI), a measure of atherosclerotic lesions, was evaluated. Mean IMT was 0.84 ± 0.07 for BDC-0, 0.94 ± 0.03 for BDC-1, 0.89 ± 0.11 for BDC-2, and 1.13 ± 0.16 for BDC-3/4 (**Figure 4A**). Max IMT was 1.11 ± 0.10 for BDC-0, 1.20 ± 0.14 for BDC-1, 1.23 ± 0.16 for BDC-2, and 1.63 ± 0.24 for BDC-3/4 (**Figure 4B**). Mean and max IMT was significantly greater for BDC-3/4 than for BDC-0 (p<0.05, p<0.005, respectively). ABI was 1.18 ± 0.06 for BDC-0, 1.08 ± 0.09 for BDC-1, 1.15 ± 0.10 for BDC-2, and 1.02 ± 0.15 for BDC-3/4 (**Figure 4C**). ABI was significantly smaller for BDC-3/4 than for BDC-0 (p<0.05).

## Discussion

These retrospective data indicate that BDC is useful in the evaluation of diabetic neuropathy and that the severity of various diabetes-related complications is associated with that of neuropathy. While no studies have contrasted BDC with atherosclerotic lesions, the data in this study indicate that BDC is associated with microvascular and macrovascular complications other than diabetic neuropathy. Also, the data in this study indicate that BDC may be useful in predicting the risk of diabetic microvascular and macrovascular complications.

The incidence of diabetic microvascular complications increases with hyperglycemia, while the incidence of macrovascular complications such as myocardial infarction has been reported to increase before the onset of diabetes and at relatively low HbA1c levels (5-7%) (8). In this study, BDC was significantly correlated with the prevalence of microvascular and macrovascular complications, despite no difference in HbA1c levels between groups. Vibration sensation cannot be performed in patients with amputated feet (9), and CVR-R is difficult to evaluate due to atrial fibrillation (10). The advantage of BDC is that it can be performed in such patients.

Previous studies on Baba classification showed an increased frequency of macrovascular complications such as myocardial infarction, stroke, and diabetic foot gangrene in patients with BDC 3-4, but there were concerns about bias due to other atherosclerotic factors (6). There have been no previous reports on the correlation between BDC and quantitative atherosclerotic factors such as mean IMT. In the present study, BDC 3-4 patients had an increased prevalence of both old myocardial infarction and old cerebral infarction. After adjustment for factors related to arterial stiffness (age, gender, BMI, history of diabetes, smoking, statins, fibrates, and systolic blood pressure), BDC was significantly correlated with mean IMT and max IMT. These data indicate that BDC can be used not only to evaluate nerve conduction velocity but also to predict atherosclerotic lesions.

Chronic hyperglycemia induces mitochondrial dysfunction, oxidative stress, and increased inflammation, all of which are likely associated with the development of diabetic neuropathy (11). On the other hand, small dense LDL-C induces inflammation *via* adipocytes and macrophages, and dyslipidemia is considered to be an influential factor in diabetic neuropathy (3). In the present study, more atherosclerotic lesions were observed in patients with advanced diabetic neuropathy which was compatible with previous report showing that there was a reverse correlation between CVR-R and carotid max IMT (10). Diabetic neuropathy is caused by damage in small nerve fibers (12), and both small nerve fibers and carotid IMT are affected by impaired glucose tolerance and vascular ischemia, which could explain the correlation between CVR-R and max IMT. In the present study, BDC was used as a new index of diabetic neuropathy to evaluate its correlation with atherosclerotic lesions, suggesting that macrovascular atherosclerosis and diabetic neuropathy may mutually aggravate the pathology. In the present study, BDC was strongly associated with the severity of microvascular and macrovascular complications. BDC-3/4 were more likely to be on hypertension and hypercholesterolemia medications and had longer diabetes duration; HbA1c and length of disease duration may correlate with the prevalence of complications, but even after adjustment for these factors The correlation was significant. On the other hand, the IMT and ABI of BDC-1 and BDC-2 participants showed little difference after adjusting for atherosclerotic factors, indicating that the severity of BDC and macrovascular complications does not necessarily match. Further study is needed to determine whether there is a direct correlation between diabetic neuropathy and macrovascular complications, or whether it is just a parallel severity of both complications.

Even in participants with BDC-0 in this study, 20.4% of them were diagnosed with diabetic retinopathy and 26.9% with nephropathy. Diabetic neuropathy is caused by small fiber neuropathy, and previous studies have reported that corneal confocal microscopy can detect small fiber neuropathy earlier than nerve conduction velocity. Corneal confocal microscopy has also been reported to detect abnormalities before the progression of diabetic retinopathy and diabetic nephropathy. The participants in this study were hyperglycemic participants who required hospitalization, and further studies are needed to evaluate the ability of BDC to detect early small fiber neuropathy in patients with shorter disease histories and less advanced complications.

This study has several limitations. First, this was a single-center, retrospective study. In addition, number of the patients in this study were limited, the generalizability may be limited. In addition, it is difficult to clinically differentiate alcoholic neuropathy from DPN, and participants with a history of alcohol consumption were excluded from the analysis. If patients with a history of alcohol consumption were included, the results might have differed from those of this study. Next, although the NCS was performed by a skilled examiner, obesity, edema, and examiner skill may have partially affected the results. In addition, since the NCS assesses indirect neurological function, we were not able to examine the correlation between the symptoms of DPN and BDC as reported in the patients in this study. Lastly, the participants in this study had severe hyperglycemia: HbA1c was as high as 10.3 ± 2.3%. It is possible that severe hyperglycemia could have affected the test results for BDC or complications. Future studies should be conducted to evaluate patients who are less affected by hyperglycemia.

In conclusion, BDC was significantly correlated with the progression of diabetic retinopathy and nephropathy. BDC was also significantly correlated with history of myocardial infarction or cerebral infarction and carotid IMT. These data suggest that BDC may be useful in predicting the presence of microvascular and macrovascular complications associated with diabetes. The data also support the idea that in patients with more severe diabetic neuropathy, we should perform further investigation of other diabetes-related complications.

## Data availability statement

The original contributions presented in the study are included in the article/supplementary material. Further inquiries can be directed to the corresponding author.

## Ethics statement

The studies involving human participants were reviewed and approved by The Institutional Review Board of Kawasaki Medical School (No. 5594-00). Written informed consent for participation was not required for this study in accordance with the national legislation and the institutional requirements.

## Author contributions

YI designed the study. YI, SN, TI, EN, MO, TKu, HT, HI, JS, YF, YK, TKi, FT, MS, SN, TM, and HK treated patients and collected data. YI analyzed the data. YI, SH, TKi, FT, MS, SN, TM, and HK contributed to discussion. KK supervised the project. YI wrote the manuscript. HK reviewed and edited the manuscript. All authors contributed to the article and approved the submitted version.

## Acknowledgments

The abstract of this report was presented at the 60th General Meeting of the Chugoku-Shikoku Regional Meeting of the Japan Diabetes Society (Hiroshima).

## Conflict of interest

HK has received honoraria for lectures, received scholarship grants, and received research grant from Novo Nordisk Pharma, Sanofi, Eli Lilly, Boehringer Ingelheim, Taisho Pharma, Sumitomo Dainippon Pharma, Takeda Pharma, Ono Pharma, Daiichi Sankyo, Mitsubishi Tanabe Pharma, Kissei Pharma, MSD, AstraZeneca, Astellas, Novartis, Kowa, Abbott. KK has been an advisor to, received honoraria for lectures from, and received scholarship grants from Novo Nordisk Pharma, Sanwa Kagaku, Takeda, Taisho Pharma, MSD, Kowa, Sumitomo Dainippon Pharma, Novartis, Mitsubishi Tanabe Pharma, AstraZeneca, Boehringer Ingelheim, Chugai, Daiichi Sankyo, Sanofi.

The remaining authors declare that the research was conducted in the absence of any commercial or financial relationships that could be construed as a potential conflict of interest.

## Publisher’s note

All claims expressed in this article are solely those of the authors and do not necessarily represent those of their affiliated organizations, or those of the publisher, the editors and the reviewers. Any product that may be evaluated in this article, or claim that may be made by its manufacturer, is not guaranteed or endorsed by the publisher.

## References

[1] Dyck PJ Herrmann DN Staff NP Dyck PJB. Assessing decreased sensation and increased sensory phenomena in diabetic polyneuropathies. Diabetes (2013) 62(11):3677–86. doi: 10.2337/db13-0352 PMC3806590 24158999

[2] Ziegler D . [Diabetic polyneuropathy]. Internist (Berl) (2020) 61(3):243–53. doi: 10.1007/s00108-020-00770-8 32086529

[3] Pop-Busui R Boulton AJM Feldman EL Bril V Freeman R Malik RA . Diabetic neuropathy: A position statement by the American diabetes association. Diabetes Care (2017) 40(1):136–54. doi: 10.2337/dc16-2042 PMC6977405 27999003

[4] Dyck PJ Karnes JL Daube J O’Brien P Service FJ. Clinical and neuropathological criteria for the diagnosis and staging of diabetic polyneuropathy. Brain (1985) 108(Pt 4):861–80. doi: 10.1093/brain/108.4.861 4075076

[5] Himeno T Kamiya H Nakamura J . Lumos for the long trail: Strategies for clinical diagnosis and severity staging for diabetic polyneuropathy and future directions. J Diabetes Investig (2020) 11(1):5–16. doi: 10.1111/jdi.13173 PMC6944828 31677343

[6] Baba M Suzuki C Ogawa. Y . Severity granding system of diabetic neuropathy in type-2 diabetes by nerve conduction study: Five-year prospective study on occurrence of diabetic foot, macroangiopathic events, and eventual death. Japanese J Clin Neurophysiol (2018) 46(2):71–7. doi: 10.11422/jscn.46.71

[7] Takahashi H Tampo H . Applying artificial intelligence to disease staging: Deep learning for improved staging of diabetic retinopathy. PLoS One (2017) 12(6):e0179790. doi: 10.1371/journal.pone.0179790 28640840 PMC5480986

[8] Sratton IM Adler AI Neil HA Matthews DR Manley SE Cull CA . Association of glycaemia with macrovascular and microvascular complications of type 2 diabetes (UKPDS 35): prospective observational study. BMJ (2000) 321(7258):405–12. doi: 10.1136/bmj.321.7258.405 PMC27454 10938048

[9] Strzalkowski ND Triano JJ Lam CK Templeton CA Bent LR. Thresholds of skin sensitivity are partially influenced by mechanical properties of the skin on the foot sole. Physiol Rep (2015) 3(6):e12425. doi: 10.14814/phy2.12425 26059035 PMC4510627

[10] Miyamoto M Kotani K Yagyu H Koibuchi H Fujii Y Konno K . The correlation between CVR-r and carotid atherosclerosis in type 2 diabetes mellitus patients with diabetic neuropathy. J Physiol Anthropol (2010) 29(4):149–52. doi: 10.2114/jpa2.29.149 20686328

[11] McAlpine CS Bowes AJ Werstuck GH . Diabetes, hyperglycemia and accelerated atherosclerosis: evidence supporting a role for endoplasmic reticulum (ER) stress signaling. Cardiovasc Hematol Disord Drug Targets (2010) 10(2):151–7. doi: 10.2174/187152910791292529 20350283

[12] Lacomis D . Small-fiber neuropathy. Muscle Nerve (2002) 26(2):173–88. doi: 10.1002/mus.10181 12210380

